# Marine Hydroquinone Zonarol Prevents Inflammation and Apoptosis in Dextran Sulfate Sodium-Induced Mice Ulcerative Colitis

**DOI:** 10.1371/journal.pone.0113509

**Published:** 2014-11-19

**Authors:** Sohsuke Yamada, Tomoyuki Koyama, Hirotsugu Noguchi, Yuki Ueda, Ryo Kitsuyama, Hiroya Shimizu, Akihide Tanimoto, Ke-Yong Wang, Aya Nawata, Toshiyuki Nakayama, Yasuyuki Sasaguri, Takumi Satoh

**Affiliations:** 1 Department of Pathology and Cell Biology, School of Medicine, University of Occupational and Environmental Health, Kitakyushu 807-8555, Japan; 2 Laboratory of Nutraceuticals and Functional Foods Science, Graduate School of Marine Science and Technology, Tokyo 108-8477, Japan; 3 Department of Welfare Engineering, Faculty of Engineering, Iwate University, Morioka 020-8551, Japan; 4 Department of Molecular and Cellular Pathology, Kagoshima University Graduate School of Medical and Dental Sciences, Kagoshima 890-8544, Japan; 5 Shared-Use Research Center, University of Occupational and Environmental Health, Kitakyushu 807-8555, Japan; 6 Laboratory of Pathology, Fukuoka Wajiro Hospital, Fukuoka 811-0213, Japan; 7 Department of Anti-Aging Food Research, School of Bioscience and Biotechnology, Tokyo University of Technology, Hachioji 192-0982, Japan; Future University in Egypt (FUE), Egypt

## Abstract

**Background and Aim:**

We previously identified an anti-inflammatory compound, zonarol, a hydroquinone isolated from the brown algae *Dictyopteris undulata* as a marine natural product. To ascertain the *in vivo* functions of zonarol, we examined the pharmacological effects of zonarol administration on dextran sulfate sodium (DSS)-induced inflammation in a mouse model of ulcerative colitis (UC). Our goal is to establish a safe and effective cure for inflammatory bowel disease (IBD) using zonarol.

**Methods and Results:**

We subjected Slc:ICR mice to the administration of 2% DSS in drinking water for 14 days. At the same time, 5-aminosalicylic acid (5-ASA) at a dose of 50 mg/kg (positive control) and zonarol at doses of 10 and 20 mg/kg, were given orally once a day. DSS-treated animals developed symptoms similar to those of human UC, such as severe bloody diarrhea, which were evaluated by the disease activity index (DAI). Treatment with 20 mg/kg of zonarol, as well as 5-ASA, significantly suppressed the DAI score, and also led to a reduced colonic ulcer length and/or mucosal inflammatory infiltration by various immune cells, especially macrophages. Zonarol treatment significantly reduced the expression of pro-inflammatory signaling molecules, and prevented the apoptosis of intestinal epithelial cells. Finally, zonarol protected against *in vitro* lipopolysaccharide (LPS)-induced activation in the RAW264.7 mouse macrophage cell line.

**Conclusions:**

This is the first report that a marine bioproduct protects against experimental UC via the inhibition of both inflammation and apoptosis, very similar to the standard-of-care sulfasalazine, a well-known prodrug that releases 5-ASA. We believe that the oral administration of zonarol might offer a better treatment for human IBDs than 5-ASA, or may be useful as an alternative/additive therapeutic strategy against UC, without any evidence of side effects.

## Introduction

Inflammatory bowel diseases (IBD), including ulcerative colitis (UC), are chronic autoimmune inflammatory disorders of the gastrointestinal tract [1], [2]. UC causes bloody diarrhea, abdominal pain and weight loss. Although UC is a complex disease orchestrated by multiple factors, and its etiology/pathogenesis is poorly understood, it is likely that immune dysregulation, mucosal barrier dysfunction and/or a loss of immunological tolerance to commensal microbiota, lead to imbalanced and elevated inflammatory cells and aberrant cytokine production [1]–[6]. Inflammatory cytokines, such as tumor necrosis factor (TNF)-α or interleukin (IL)-1β, have been implicated in the pathogenesis of UC [3]–[6].

Sulfasalazine, a prodrug composed of 5-aminosalicylic acid (5-ASA) and sulfapyridine, has been used as a standard-of-care in UC for decades, but is a double edged sword because it generates excessive oxidative stress, resulting in severe adverse symptoms, such as blood disorders, hepatotoxicity, ulcerogenic potential and hypospermia and male infertility [7], [8]. Other therapies or combinations of drugs, including novel molecular targeted drugs or antigen-specific immunotherapy, have been of no or very limited benefit, with potential severe side effects [1], [2]. UC also predisposes patients to subsequent colorectal cancer and/or the need for intestinal surgeries [1], [2], [9].

In this context, promising safe and effective drugs are needed for these vulnerable UC patients. In fact, approximately 30–50% of IBD patients seek symptom relief and an improved quality of life, and complementary and alternative medicine (CAM) has often been administered in addition to their primary medications [10]. The variety of CAM therapies includes: (i) hypnosis, (ii) acupuncture, (iii) megadoses of vitamins and minerals, (iv) prebiotics, (v) probiotics and (vi) herbal therapies [10]–[12].

In the Asian-Pacific region, various types of seaweed have been used, particularly as foodstuff, and as folk medicine to maintain health throughout the ages. More recently, some species of seaweed and seaweed-containing ingredients have become a popular and easily recognized food around the world. In order to obtain a novel inhibitor of inflammation (carrageenan-induced edema in mice) from marine-derived biomass products, we screened the extracts of 150 marine species from around the shore of the Japanese mainland, and found that a crude extract of *Dictyopteris undulata* had the most potent inhibitory effects [13]. Our subsequent experiments showed that the active compound in this extract was zonarol, based on the nuclear magnetic resonance (NMR) data after bioassay-guided purification from the crude extract of *Dictyopteris undulata*. Originally, this sesquiterpene hydroquinone had been reported as a fungitoxic compound by Fenical and Cimino in the 1970's, and had been derived from *Dictyopteris zonarioides* (synonymous with *Dictyopteris undulata*) [14], [15]. The absolute chemical structure of the sesquiterpene was elucidated in 1986 [16]. Other pharmacological actions of zonarol have been reported, such as antioxidant effects [17], phospholipase inhibition [18], feeding deterrents against abalone [19] and algicidal effects [20]. To the best of our knowledge, there are no data regarding the *in vivo* effects of the marine hydroquinone, zonarol, especially with regard to its anti-inflammatory effects.

In order to clarify the pharmacological actions of this compound, the present study examined the anti-inflammatory actions of zonarol purified from *Dictyopteris undulata* in both experimental animals and a cultured cell line. In particular, we examined the protective roles of zonarol in dextran sulfate sodium (DSS)-induced colon injury using young male Slc:ICR mice, since this model demonstrates the progression of inflamed mucosal lesions with erosion to ulcer formation, the infiltration of various inflammatory cells and accelerated production of multiple inflammatory and/or pro-inflammatory mediators, reminiscent of human UC. We examined the potential of using zonarol as an alternative/additive CAM treatment for UC.

## Materials and Methods

### Preparation of crude extract from the brown algae, *Dictyopteris undulata*

Brown algae (*Dictyopteris undulata*) was collected from the intertidal area in Shizuoka and Kanagawa prefectures, Japan, in 2013 (35.140612, 139.651754 or 34.709215, 138.984292 or 34.631562, 138.899008 by the GPS coordinates). For these locations/activities, no specific permissions were required. The fresh alga body (3.0 kg) was drained off on a paper towel, and then extracted with five volumes of methanol for five days at room temperature. The extract obtained by two times procedures was filtered, evaporated and freeze-dried to give dark green powder (108.0 g). The powder was stored at −20°C until use for *in vivo* and *in vitro* experiments as a crude extract.

### Purification and structural determination of zonarol

The active component of the crude extract was fractionated by partition and column chromatography techniques based on our preliminary data regarding the suppressive activity of the extract against carrageenin-induced paw edema in mice. The fraction with the activity was used for further fractionation until a single compound was detected based on high performance liquid chromatography (HPLC) data. The structure of the purified compound was then determined based on spectral data from NMR experiments. The NMR spectra in MeOD were obtained on a Bruker AV-400 spectrometer (Bruker, Tokyo, Japan). Liquid Chromatography–Mass Spectrometry (LC/MS) was performed using a GC-2010 instrument (SHIMADZU, Kyoto, Japan). Confirmation of the purity of the compound was based on the HPLC analysis and spectral data.

### Animals and the ulcerative colitis (UC) model

Experiments were performed using five-week-old male Slc:ICR mice after a one-week acclimatization period. The mice weighed 30–35 g, were purchased from Japan SLC (Hamamatsu, Japan) and were maintained in a temperature and light-controlled facility with free access to standard rodent chow and water. In the current UC model, we subjected the five-week-old Slc:ICR mice in the DSS group to the administration of 2% DSS in drinking water for 14 days. The Slc:ICR mice in the positive control and zonarol groups were also allowed to drink 2% DSS water *ad libitum* for 14 days, and at the same time, were given 5-aminosalicylic acid (5-ASA; Sigma-Aldrich Chemical Co., Ltd.; St. Louis, MO, USA) at a dose of 50 mg/kg, and/or zonarol at doses of 10 and 20 mg/kg orally once a day for 14 days. Normal control ICR mice received a sham treatment. The body weight (BW) and food and water intake were assessed every day, and the organ weights and colon length were determined when the mice were killed. Food consumption was determined using metabolic cages obtained from SUGIYAMA-GEN Co., Ltd. (Tokyo, Japan) [21]. Six weight-matched animals from each group were analyzed (n = 30 in total). The experimental procedure is summarized in Figure S1, as a schematic representation.

The disease activity index (DAI) was calculated by grading on a scale of 0 to 4 using the following parameters: loss of BW (0, normal; 1, 0–5%; 2, 5–10%; 3, 10–20%; 4, >20%), stool consistency (0, normal; 2, loose stools; 4, watery diarrhea) and the occurrence of gross blood in the stool (0, negative; 4, positive). The combined DAI scores were determined by two independent investigators (Sohsuke Yamada and Tomoyuki Koyama) blinded to the study results [21]. There were no cases of disagreement in this DAI score. On day 15 after the induction of UC or sham-treatment, the mice were euthanized by exsanguination under general anesthetization with spontaneous inhalation of isoflurane (Mylan Inc., Canonsburg, PA, USA). The peritoneal cavity was then opened, blood samples were taken from the inferior vena cava, and several tissues, including intestines and/or the spleen, were excised (n = 30 in total). In all animals, the colon from the ileocecal junction to the anus was excised, cut open lengthwise or cut into small pieces (n = 30 in total), and used for various experiments, as described below.

### Ethics

The animal studies were conducted according to the 2006 guidelines entitled “Notification No. 88 of the Ministry of the Environment in Japan and Guidelines for Animal Experimentation of Tokyo University of Marine Science and Technology” with the approval of the Animal Care and Use Committee of Tokyo University of Marine Science and Technology. The investigation conformed to the Guide for the Care and Use of Laboratory Animals published by the US National Institutes of Health (NIH Publication No. 85-23, revised 1996).

Our field studies did not involve endangered or protected species, and no specific permissions for any locations/activities were required.

### Histopathology

Colon specimens were stained with hematoxylin and eosin (H&E), Alcian blue or periodic acid-Schiff (PAS) stain, or were used for immunohistochemistry (IHC) preparations in sequential sections, after fixation in 15% neutral buffered formalin for 24 hr [22]–[26]. The analyses were performed in DSS-induced inflamed intestines in all experiments, whereas non-treated colons served as normal controls.

Colons embedded in paraffin for histological examinations were cut systematically in sequential longitudinal sections of 4-µm thickness using a sliding microtome (Leica SM2010R, Leica Microsystems, Wetzler, Germany). For the histological analyses of the large intestine, images of H&E (n = 30 in total) and specially stained sections or IHC sections (n = 18 in total) were captured and quantified using the NanoZoomer Digital Pathology Virtual Slide Viewer software program (Hamamatsu Photonics Corp., Hamamatsu, Japan). H&E-stained longitudinal sections were graded by two independent pathologists (Sohsuke Yamada and Hirotsugu Noguchi) blinded to the physical outcome and other biological and pathological data for each sample, using a scoring system to evaluate the neutrophil infiltration (0–3), lymphocyte infiltration (0–3), erosion to ulceration (0–4) and crypt destruction or loss (0–3) [21]. The maximum sum of the scores for a given section was 13. The DAI and histological scores were from the same mouse [21]. The mean number or average size of goblet cells per crypt was quantified by Alcian blue or PAS staining in 10 randomly selected fields per section (original magnification: ×400) [22]–[24].

### Analyses of inflammatory responses to DSS-induced UC injury by immunohistochemistry (IHC) and double-immunofluorescence (IF) staining

One representative sequential section per mouse was prepared for IHC staining, and was captured and evaluated by a NanoZoomer Digital Pathology Virtual Slide Viewer (Hamamatsu Photonics Corp.) to avoid potential bias [22]–[25].

To evaluate the severity of DSS-induced UC on day 15, we determined the intensity of inflammation using a polyclonal rabbit anti-human CD3 antibody (1∶1; Dako, Glostrup, Denmark), a rat anti-mouse Mac-2 monoclonal antibody (1∶500; Cedarlane Laboratories Ltd., Burlington, Ontario, Canada) or a rat anti-mouse Ly-6G antibody (Gr-1; 1∶500; Birmingham, AL, USA) [22]–[26]. We counted the number of positive T-lymphocytes, macrophages or neutrophils in 10 randomly selected fields of inflamed mucosal areas per section (original magnification: ×400) [22], [23].

To assess the degree of infiltration of pro-inflammatory mucosal macrophages by double-immunofluorescence (IF), the injured colonic mucosa was labeled with a mouse monoclonal tumor necrosis factor (TNF)-α antibody (1∶50; Abcam, Burlingame, CA, USA) and rat monoclonal Mac-2 antibody (1∶500; Cedarlane Laboratories Ltd.), visualized with goat anti-mouse IgG antibodies conjugated with Alexa Fluor Dyes (*red-stained*) and goat anti-rat IgG and IgM antibodies conjugated with Alexa Fluor Dyes (*green-stained*) (Invitrogen Corp., Camarillo, CA, USA), respectively, and viewed by confocal laser scanning microscopy (LSM5 Pascal Exciter; Carl Zeiss, Oberkochen, Germany) (original magnification: ×400) [22], [24]. We applied the HistoMouse *Plus* Kit (Invitrogen) to block endogenous mouse IgG [22]–[25]. Furthermore, in order to analyze the expression of inducible nitric oxide synthase (iNOS) in the inflamed mucosal lesions, an anti-iNOS rabbit polyclonal antibody (1∶200; BD Biosciences, San Jose, CA, USA) was applied, and we quantified the positive areas in 10 randomly selected fields per section (original magnification: ×400) [22]–[26].

For IHC or IF studies, we examined one section from each of six mice per experimental group (n = 18 in total, respectively). All histological and IHC or IF slides were evaluated by two independent observers (certified pathologists: Sohsuke Yamada and Hirotsugu Noguchi) who were blinded to the physical outcome or other biological and pathological data for each sample. In case of disagreement, a consensus score was determined by a third board-certified pathologist (Yasuyuki Sasaguri). The agreement between observers was excellent (>0.9) for all sections investigated, as measured by interclass correlation coefficient.

### Terminal deoxynucleotidyl transferase end-labeling (TUNEL)

TUNEL assays were performed using an *In Situ* Cell Death Detection Kit, POD (Roche Applied Science, Mannheim, Germany) [22]–[25]. Additionally, colon sections were stained with a rabbit polyclonal anti-mouse cytokeratin 20 (CK20) antibody (Abcam, Cambridge, England) to highlight the epithelial lining of the colonic mucosa. DSS-injured colons on day 15 were labeled with anti-fluorescein antibodies (*brown-stained*) (TUNEL POD; Roche Applied Science), or fluorescein-conjugated TUNEL reaction mixture (*green-stained*) (Roche Applied Science) and a rabbit polyclonal anti-CK20 antibody (1∶200; Abcam). For the latter, the staining was visualized with donkey anti-rabbit IgG antibodies conjugated with Alexa Fluor Dyes (*red-stained*) (Invitrogen) by confocal laser scanning microscopy (LSM5 Pascal Exciter) (original magnification: ×400) [22], [24]. For a quantitative analysis, we counted the TUNEL^+^ lining epithelial cells (*brown-stained*) in 100 randomly selected crypts per section (original magnification: ×400) [22]–[25].

### Enzyme-linked immunosorbent assay (ELISA) for TNF-α and interleukin (IL)-6

The levels of serum TNF-α and interleukin (IL)-6 in the DSS-induced UC model on day 15 were measured using an ELISA kit according to the manufacturer's instructions (R&D Systems, Minneapolis, MN, USA) [22], [24].

### Cell culture

RAW264.7 cells, obtained from the American Type Culture Collection (ATCC; Manassas, VA, USA) were grown in Dulbecco's modified Eagle's medium (DMEM) containing 10% fetal bovine serum (FBS), 100 µg/mL streptomycin and 100 U/ml penicillin (Life Technologies; Carlsbad, CA, USA) in 100-mm Petri dishes (BD Falcon; Franklin Lakes, NJ, USA). The final concentration of DMSO in the culture medium was 0.1%. These RAW264.7 cells are often used as an *in vitro* model of macrophage activation. The cells were seeded in 24-well plates at a density of 1×10^5^ cells/cm^2^ in normal DMEM medium. The next day, after washing the cells with PBS, the medium was replaced with 500 µL of serum-free DMEM containing 0.02% BSA and 10 µg/mL lipopolysaccharide (LPS) (L8274, *E. coli* 026:B6-derived) (Sigma-Aldrich Chemical Co. Ltd.) with or without zonarol at the indicated concentrations. After a 24-h incubation at 37°C, the cells were subjected to the following procedures: the 3-(4,5-dimethylthiazol-2-yl)-2,5-diphenyl tetrazolium bromide (MTT) assay to assess viability, nitric oxide (NO) assay and reverse transcription-polymerase chain reaction (RT-PCR), as described elsewhere [27]–[29].

### MTT and NO assays

The viability and NO production of sister cultures of the cells were determined using the MTT assay (DOJINDO, Tokyo, Japan) and Griess reagent (Invitrogen; Carlsbad, CA, USA), respectively. The generation of NO was determined by measuring the nitrite accumulation in the medium with modified Griess reagent. The culture supernatant and Griess reagent were mixed and incubated for 5 min, and subsequently, the absorption was determined at 540 nm. Sodium nitrite (NaNO_2_) was used to generate a standard curve for quantification [28], [29].

### Reverse transcriptase-polymerase chain reaction (RT-PCR)

Total RNAs were extracted with the Trizol reagent (Invitrogen) from the RAW264.7 mouse macrophage cell line after 24 h of treatment with zonarol (2 µM) and/or LPS. All procedures were performed as described previously [22]–[26]. RNase-free conditions were used to prevent mRNA degradation. First-strand cDNA was synthesized with Superscript II RT (Invitrogen) using random primers, according to the manufacturer's instructions. One one-hundredth of the cDNA was used for each PCR reaction. The cycling conditions were as follows: 50°C for 2 min, 95°C for 10 min followed by 45 cycles of 95°C for 15 s and 60°C for 1 min. The following mouse pairs of primers specific for β-actin, mIL-1β, mIL-6 and miNOS were used: 5′-ATC CGT AAA GAC CTC TAT GC-3′ (forward) and 5′-AAC GCA GCT CAG TAA CAG TC-3′ (reverse) for β-actin, 5′-CAA CCA ACA AGT GAT ATT CTC CAT-3 (forward) and 5′-GAT CCA CAC TCT CCA GCT GCA GGG-3′ (reverse) for mIL-1β, 5′-GGA GAC TTC ACA GAG GAT AC-3′ (forward) and 5′-CCA GTT TGG TAG CAT CCA TC-3′ (reverse) for mIL-6, and 5′-CAG CTG GGC TGT ACA AAC CTT-3′ (forward) and 5′-CAT TGG AAG TGA AGC GTT TCG-3′(reverse) for miNOS, resulting in 287-bp, 152-bp, 212-bp and 95-bp RT-PCR products, respectively. At the completion of the PCR, 10 µL of PCR products were mixed with 2 µL of loading buffer and electrophoresed in 1.5% agarose gel in the presence of 0.5 µg/mL of ethidium bromide, and were visualized with a UV transilluminator.

### Statistical analysis

**Figure 1:**
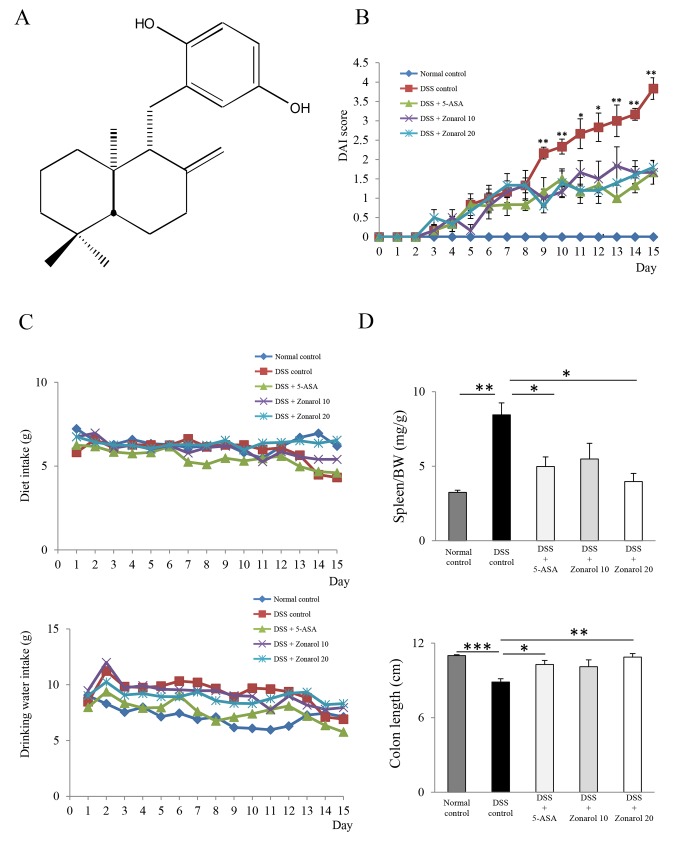
Zonarol suppresses DSS-induced UC in Slc:ICR mic. **A**) The chemical structure of zonarol, a sesquiterpene para-hydroquinone. **B**) The DAI scores were dramatically increased in DSS positive control mice compared with those in the other groups of mice treated with zonarol at 10 mg/kg or 20 mg/kg or 5-ASA 50 mg/kg from day 9 to 15. We observed no apparent difference in the DAI between the mice treated with zonarol and 5-ASA. **C**) There were no remarkable differences in the food and drinking water intake between the different groups of mice throughout the experimental period (n = 6 mice per group). However, the water intake in the DSS groups tended to be gradually and slightly decreased after DSS administration. **D**) On day 15 post-DSS administration (n = 6 mice per group), the spleen/BW ratio in the DSS control group was significantly elevated, whereas that in the zonarol 20 mg/kg and 5-ASA groups was not. Correspondingly, the colon length was significantly shortened in the DSS mice, compared with the normal control mice on day 15 in the present UC model (n = 6 mice per group), whereas zonarol at 20 mg/kg and 5-ASA treatment both significantly suppressed the colon shortening. The values are the means ± SE. *P<0.05, **P<0.001, ***P<0.0001.

**Figure 2:**
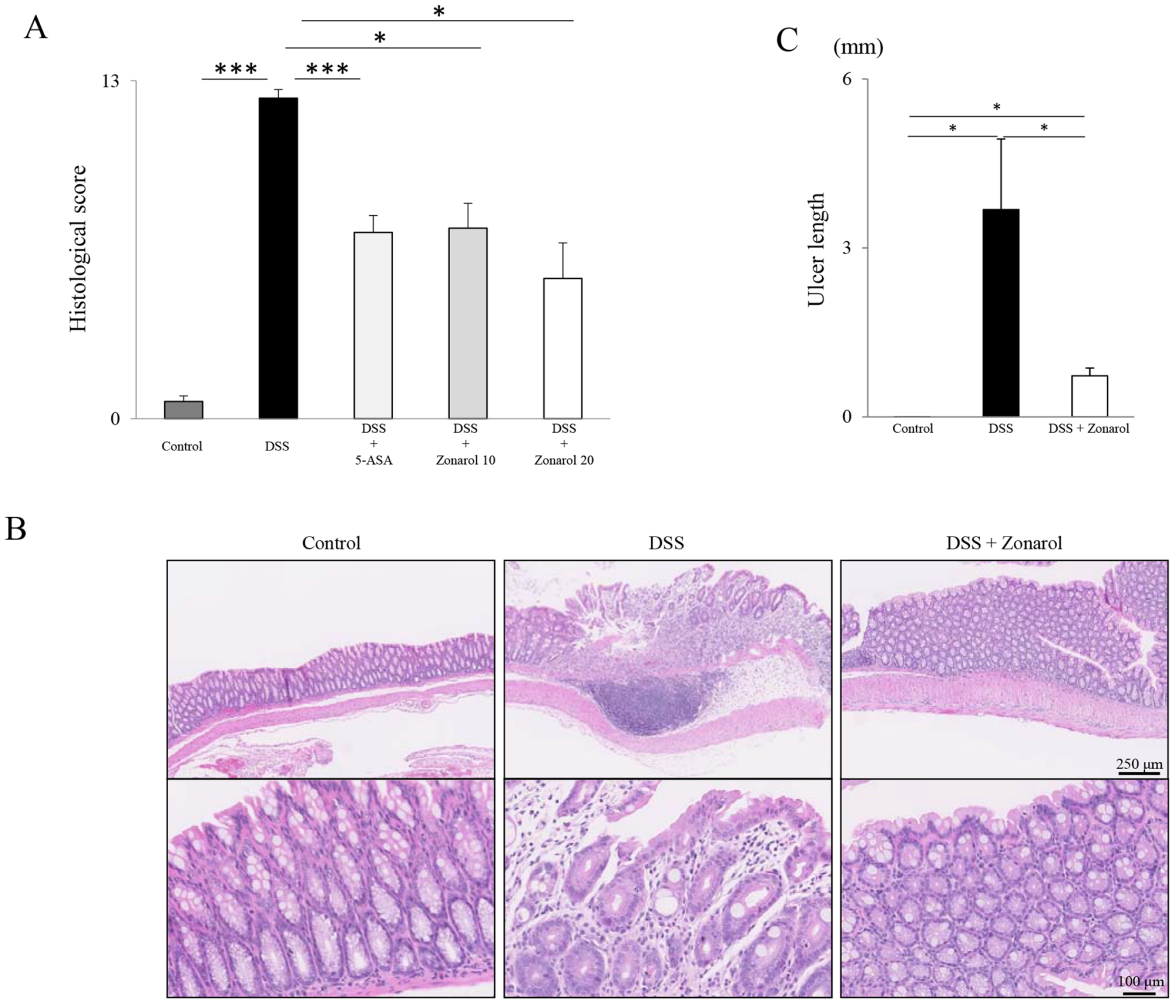
Zonarol histologically dampens DSS-induced UC in Slc:ICR mice. **A**) DSS administration resulted in a significantly higher histological score, characterized by acute and chronic inflammatory cell infiltration, erosion and/or occasional ulcer formation and crypt loss, compared to normal control mice (n = 6 mice per group). Both 5-ASA and zonarol at 10 and 20 mg/kg significantly reduced the inflammatory histological score on day 15. **B**) In the representative H&E-stained colon sections, fewer and smaller colonic ulcers were detected in the mice in the 20 mg/kg zonarol group, compared with the DSS positive control mice after DSS stimulation for 14 days (n = 6 mice per group). Normal control mice showed no remarkable changes in the colon on day 15. Upper panel: low-power view (Scale bar = 250 µm). Bottom panel: high-power view (Scale bar = 100 µm). **C**) The sum of the histological ulcer length in the mice in the 20 mg/kg zonarol was significantly larger than that in the normal control mice on day 15 post-DSS administration, but was smaller than that in the DSS positive control mice (n = 6 mice per group). The values are the means ± SE. *P<0.05, ***P<0.0001.

**Figure 3:**
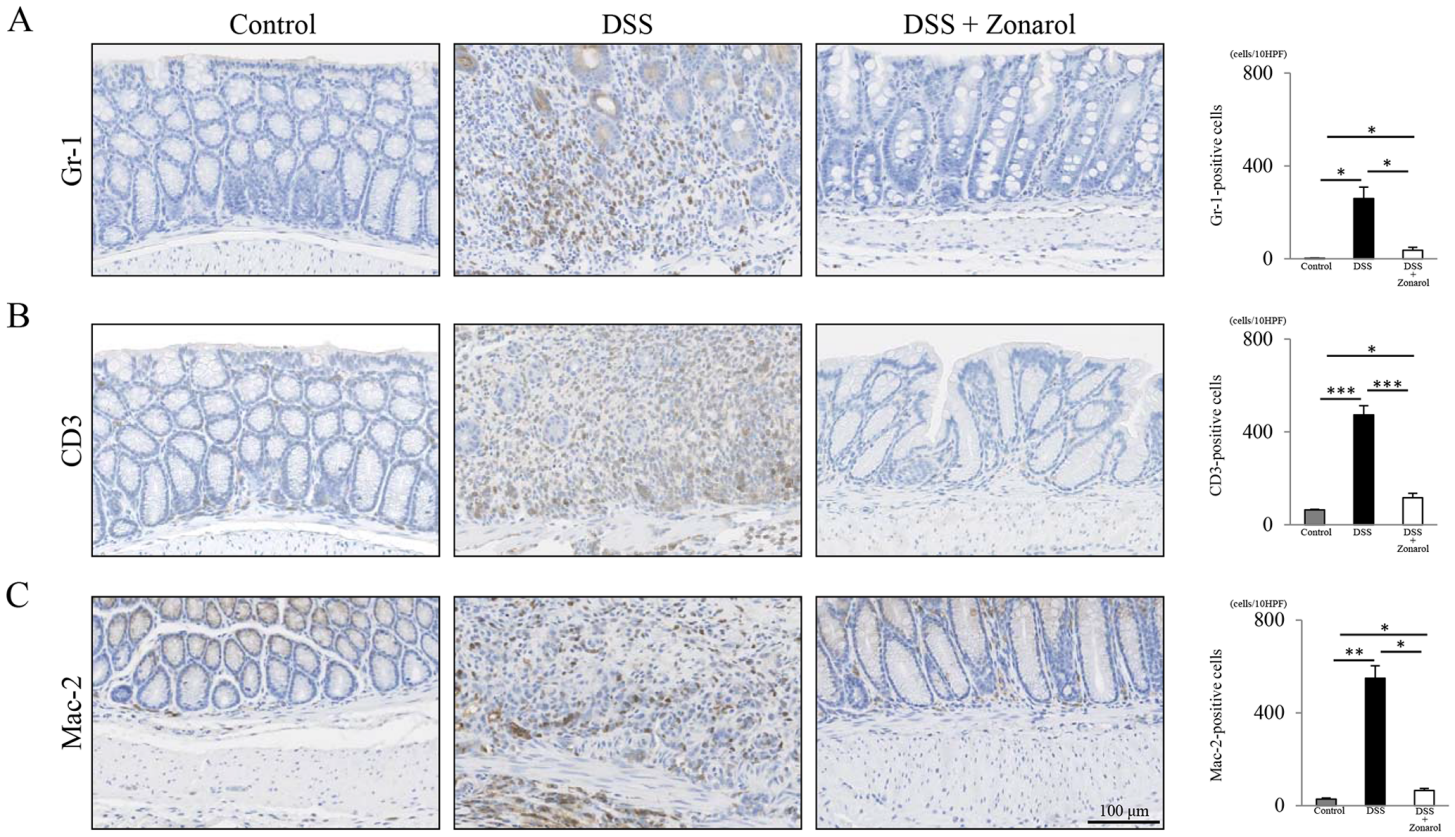
Zonarol represses the inflammatory responses in the subacute and chronic phases after DSS-administration in UC mice. **A**) IHC for Gr-1 showed a significantly greater decrease in the numbers of accumulated neutrophils (i.e., subacute inflammatory cells) in the modestly injured colonic mucosa in the mice from the 20 mg/kg zonarol group compared to the positive control mouse colons on day 15 post-DSS administration (n = 6 mice per group). Fewer than 10 neutrophils per 100 crypts were noted in sham-treated normal control mice. **B, C**) IHC for CD3 revealed that the modestly-injured colons in mice from the 20 mg/kg zonarol group had significantly fewer infiltrating T-lymphocytes, especially around the crypts in the lamina propria, compared with the DSS positive control mice on day 15. In addition, Mac-2 staining showed that there were significantly fewer macrophages per mucosa in the mice in the 20 mg/kg zonarol group than in the mice in the positive control group. In contrast, untreated normal control animals had markedly fewer chronic inflammatory cells (T-lymphocytes and macrophages) compared to the mice in the 20 mg/kg zonarol group. Scale bar = 100 µm. Values are the means ± SE. *P<0.05, **P<0.001, ***P<0.0001.

**Figure 4:**
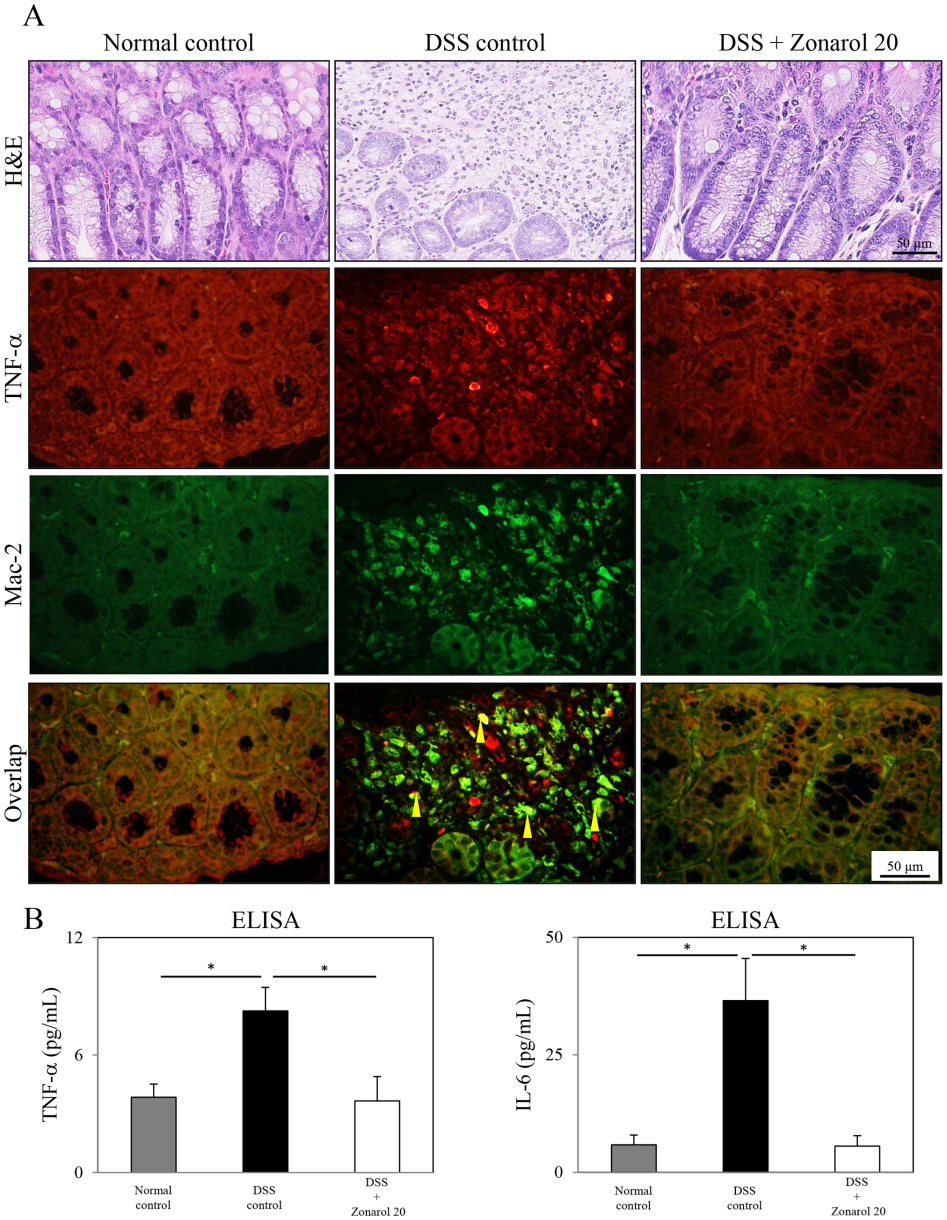
Zonarol suppresses the expression of pro-inflammatory signaling factors, such as TNF-α and IL-6, in mice with DSS-induced UC. **A**) The IF study revealed that the number of TNF-α^+^ (*red-stained*) mucosal cells was obviously higher in the injured colons (H&E stain) of the DSS positive control mice than in the modestly inflamed colons (H&E stain) of the 20 mg/kg zonarol group (n = 6 mice per group). Additionally, the number (overlap) of both TNF-α^+^ (*red-stained*) and Mac-2^+^ (*green-stained*) macrophages in the inflamed lamina propria was also significantly more increased in the positive control mice than in the zonarol-treated mice 15 days after the DSS administration based on the IF results. Sham-treated normal control animals showed no remarkable changes, and carried no or very few cells with overlapping staining. **B**) Corresponding to these IHC and IF data, an ELISA demonstrated that the serum levels of not only TNF-α, but also IL-6, were significantly higher in the DSS positive control mice than in both the zonarol-treated mice and the normal control mice (n = 6 mice per group). Scale bar = 50 µm. The values are the means ± SE. *P<0.05.

**Figure 5:**
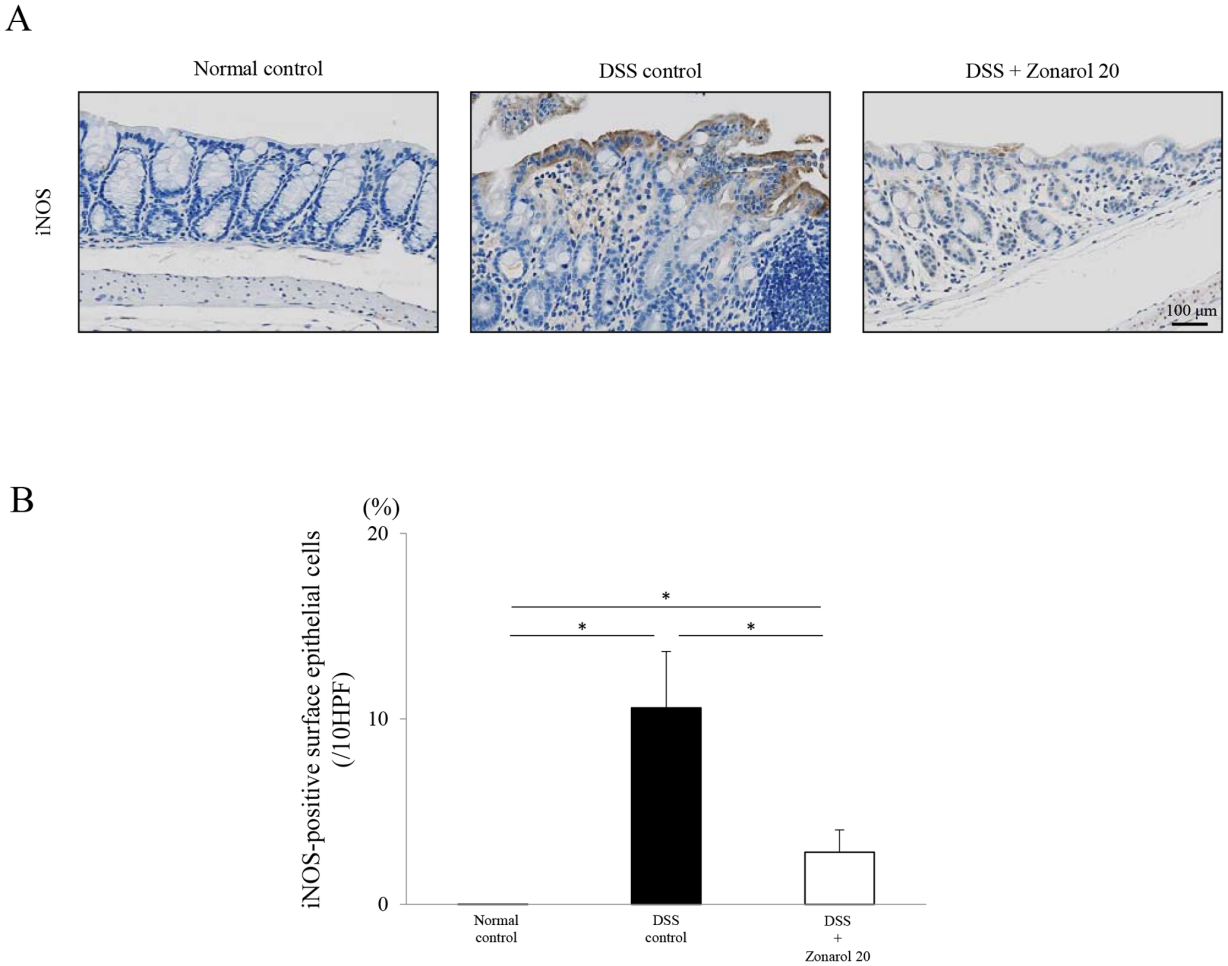
Zonarol suppresses the expression of pro-inflammatory signaling molecules, such as iNOS, in mice with DSS-induced UC. A, B ) IHC of iNOS showed that, in established DSS-induced UC, the iNOS expression level (i.e., the iNOS-positive area) was substantially upregulated, especially in the surface colonic epithelium of the DSS positive control mice, but not in the mice treated with zonarol, on day 15 post-DSS administration (n = 6 mice per group). No apparent iNOS-positive areas were seen in the sham-treated normal control mice. Scale bar = 100 µm. The values are the means ± SE. *P<0.05.

**Figure 6:**
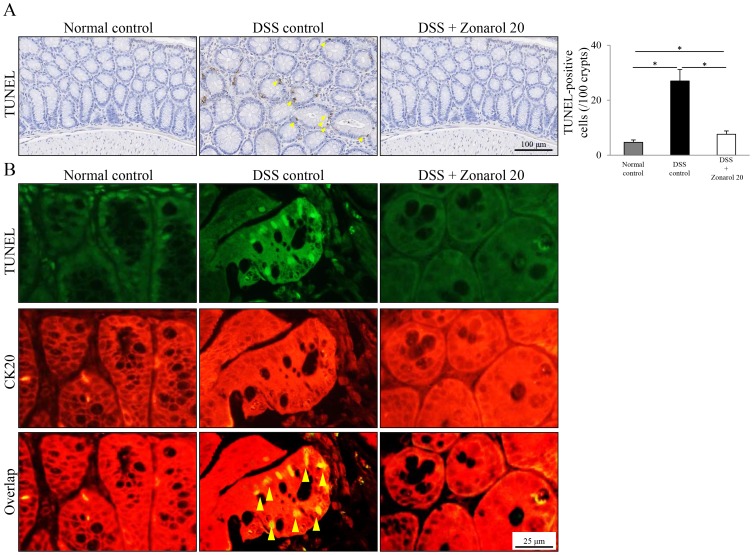
Zonarol reduces the apoptotic activity of the colonic epithelium in mice with DSS-induced UC. **A**) A small but substantial number of apoptotic epithelial cells (TUNEL staining) in crypts were identified in each group of mice 15 days after the administration of DSS (n = 6 mice per group). The number of TUNEL^+^ (*green-stained* in nuclei) large intestinal epithelial cells in the zonarol-treated mice was significantly larger than that in the normal control mice on day 15 post-DSS administration, but was smaller than that in the DSS positive control mice. **B**) Double-IF staining confirmed that these TUNEL^+^ apoptotic cells (*green-stained* in nuclei) were CK20^+^ colonic epithelial cells in the crypts (*red-stained* in cytoplasm). Scale bars = 100 µm (medium-power view); and 25 µm (high-power view). The values are the means ± SE. *P<0.05.

**Figure 7:**
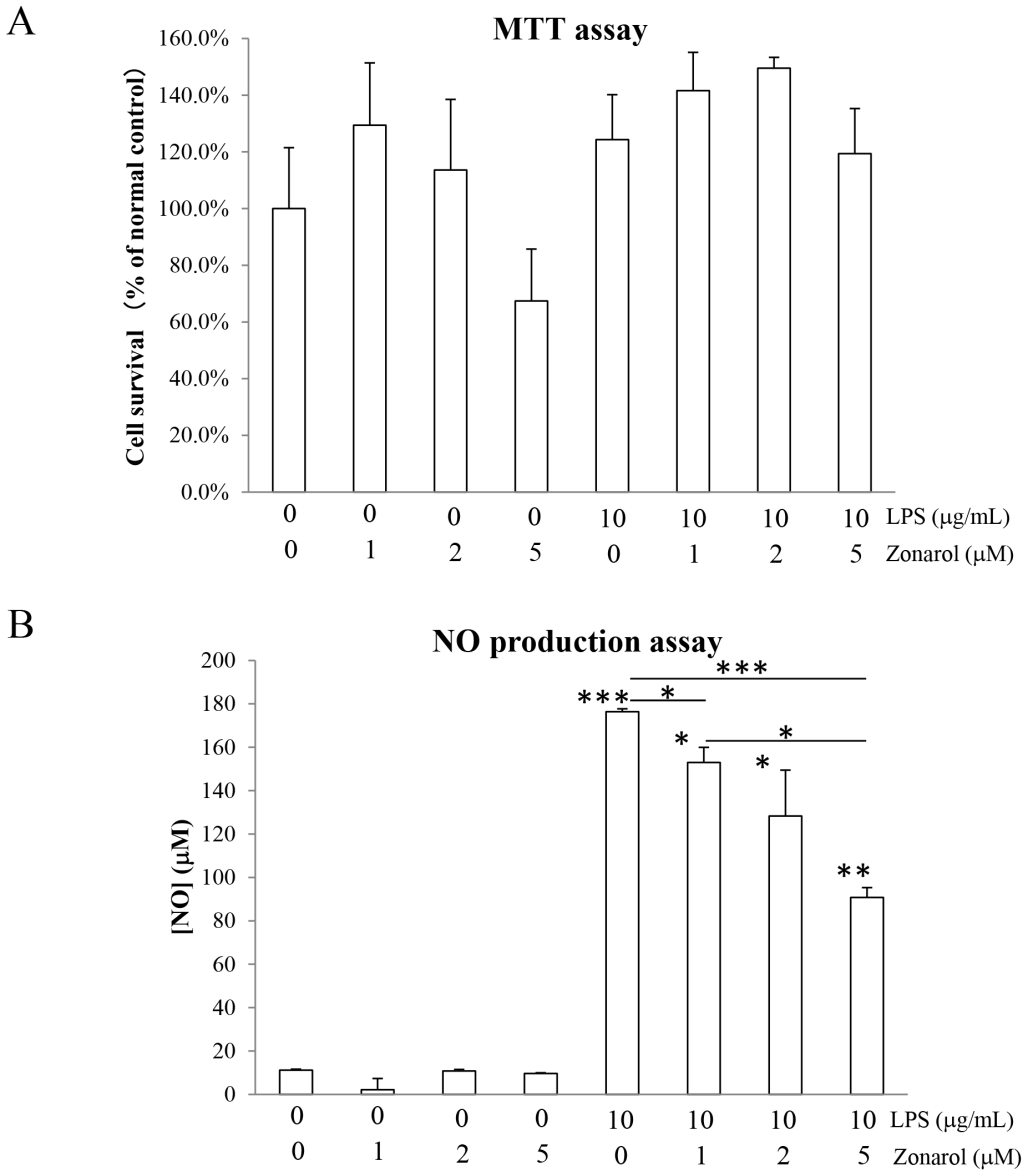
Zonarol inhibits NO production without any effects on the survival of cultured RAW264.7 cells after LPS stimulation. **A**) **Cell survival.** RAW264.7 cells were incubated for 24 h with the various combinations of zonarol and LPS, as indicated in the figure. The cell viability was determined using the MTT assay. Note that there were no significant differences in the viability of any of the treated cells compared to the normal or positive control cells. **B**) **NO production.** The LPS-induced NO production was inhibited by zonarol in a dose-dependent manner in RAW264.7 cells. The RAW264.7 cells were treated with combinations of LPS and zonarol for 24 h, and the NO concentrations were determined using the Griess reagent. The values are the means ± SD from four separate measurements. * and ** Significant differences (*P*<0.05 and 0.001, respectively) compared with the 0 µg/mL LPS- and 0 µM zonarol-treated cells (normal control) or the 10 µg/mL LPS- and zonarol 0 (positive control) or 1 µM-treated cells.

**Figure 8:**
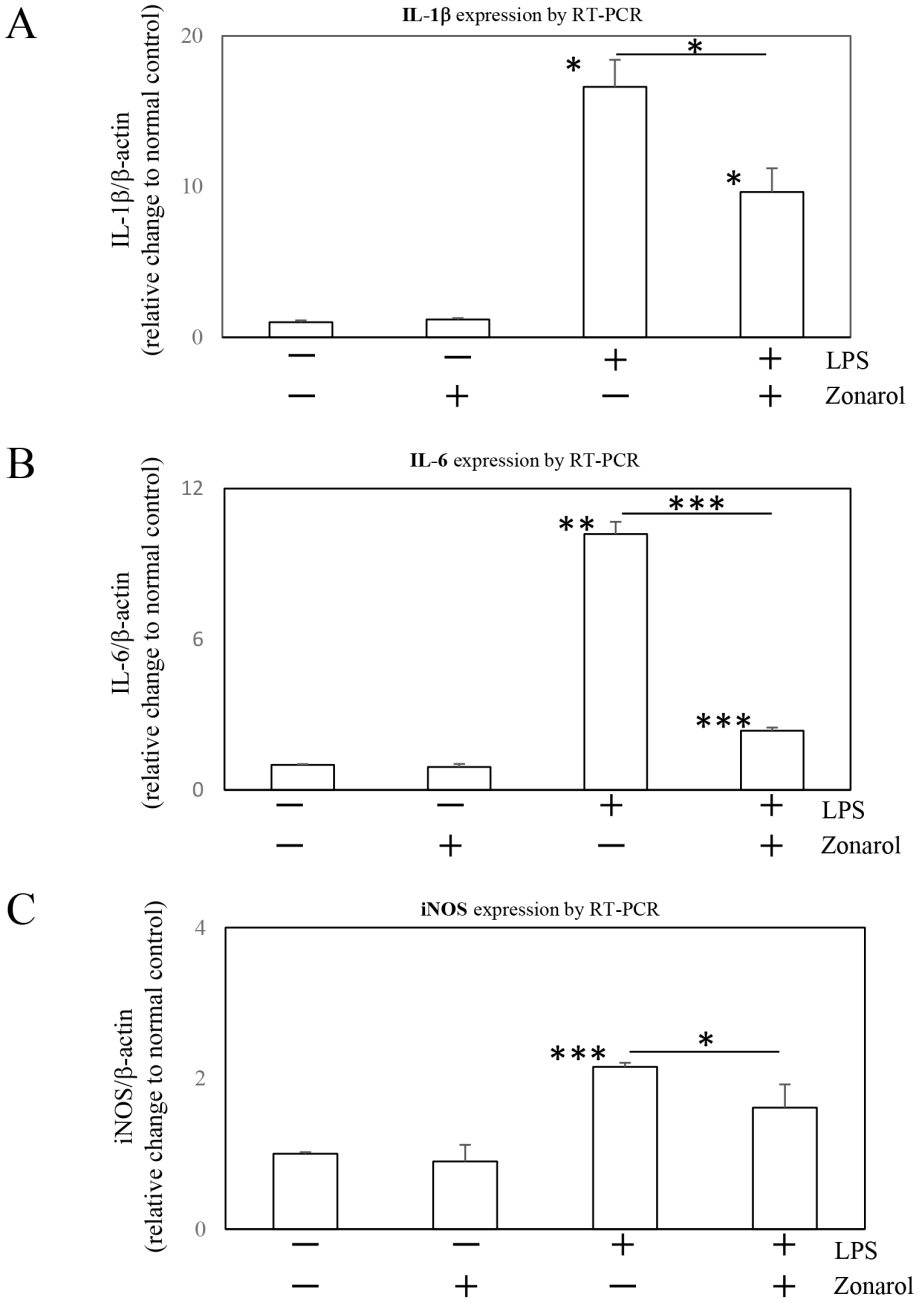
Zonarol downregulates the expression of immune system mediators in cultured RAW264.7 cells after LPS stimulation. A, B, C ) RAW264.7 cells were left untreated or were treated with zonarol (10 µM), LPS (10 µg/ml) or both for 24 h. The levels of IL-1β, IL-6 and iNOS mRNA were determined by RT-PCR. In the absence of LPS, the cells had very low expression levels of these genes; whereas in the presence of LPS, there was a significant increase in their expression. Zonarol at a concentration of 2 µM significantly decreased the mRNA levels of IL-1β (A), IL-6 (B) and iNOS (C). The values are the means ± SD from four separate measurements and were normalized to the β-actin expression (RT-PCR). *, **, and *** Significant differences (*P*<0.05, 0.001, and 0.0001, respectively) compared with the 0 µg/mL LPS- and 0 µM zonarol-treated cells (normal control).

The results are expressed as the means ± SE (*in vivo*) or ± SD (*in vitro*). Significant differences were analyzed using Student's *t*-test, Welch's *t*-test or a one-way ANOVA (analysis of variance), where appropriate. In all cases when the ANOVA methodology was employed for non-parametric data (Figs. 1–8), except for results on a carrageenan-induced paw edema mouse model (Fig. S3), Tukey's multiple comparison *post-hoc* test was used [22]–[26]. Values of *P*<0.05 were considered to be statistically significant.

## Results

### Evaluation of the chemical properties of purified zonarol

Zonarol (Fig. 1A) was isolated from the crude extract of seaweed *Dictyopteris undulata* as one of the most potent inhibitors of inflammation in mice. To identify the fraction containing the inhibitor, a fractionation procedure was performed using the crude extract (Fig. S2). The final separation step on an Octadecyl Silyl (ODS) column (250×20 mm i.d.) by HPLC (85% aqueous MeOH, 10 mL/min) gave compound **1** (1.1% of yield) as a single peak at 17.0 min, which was detected by UV (280 nm). The purified compound showed significant effects in an assay in mice (Fig. S3). A LCMS analysis of **1** gave *m/z* 314 as a molecular ion [M+H]^+^. Finally, the chemical structure of **1** was identified as zonarol based on these and the proton and carbon NMR data (Fig. S4).

### Zonarol grossly suppresses DSS-induced UC in Slc:ICR mice

To determine the *in vivo* effects of the zonarol extract, Slc:ICR mice were exposed to 2% DSS in their drinking water for 14 days. Since gross bleeding in stool and diarrhea were early signs that occurred around day 9 after starting DSS administration, the BW loss compared to the group of normal control was clearly greater the DSS control group mice from day 11 of the present UC model, but the difference was less than 5%. Therefore, the DAI scores were significantly and dramatically increased in DSS-positive control mice during from day 9 to 15 in the UC model (day 15: DSS 3.83±0.28 vs. Normal 0±0; *P*<0.0001), compared with the other groups of mice treated with zonarol at 10 mg/kg (1.67±0.30) or 20 mg/kg (1.80±0.18) or with 5-ASA (1.67±0.30) (*P*<0.001, respectively) (Fig. 1B). We observed no apparent BW change in the mice treated with zonarol and 5-ASA.

In addition, there was no remarkable change in the food and drinking water intake between the groups of mice throughout the experimental period in the present UC model (Fig. 1C). However, the drinking water intake in the DSS groups tended to be gradually and slightly decreased after DSS administration (Fig. 1C). In contrast, the spleen/BW ratio (Fig. 1D) in the DSS control group was significantly elevated (DSS 8.45±0.80 mg/g vs. Normal 3.25±0.14 mg/g; *P*<0.001), whereas that in the zonarol 20 mg/kg (3.97±0.55 mg/g) and 5-ASA (4.98±0.65 mg/g) groups were significantly suppressed on day 15 after DSS injury (*P*<0.05, respectively) (Fig. 1D). However, the thymus/BW ratios did not change (data not shown). Correspondingly, the colon length was significantly shortened in the DSS group compared with the normal control mice on day 15 (DSS 8.87±0.26 cm vs. Normal 11.00±0.07 cm; *P*<0.0001), whereas the lengths in the zonarol 20 mg/kg (10.88±0.28 cm) and 5-ASA (10.28±0.33 cm) treatment groups exhibited significantly suppressed colon shortening (*P*<0.001 and *P*<0.05, respectively) (Fig. 1D).

On the other hand, there was no remarkable change in mortality between the normal control and the DSS-induced colitic mice within 15 days (data not shown), but each group of animals was also free of complications, and none of the mice died during the study period.

### Zonarol decreases the DSS-induced UC-associated histological changes in Slc:ICR mice

Corresponding to the above gross findings, including the DAI scores, DSS administration significantly enhanced the inflammation of the colon compared to the normal control mice, as indicated by several parameters, such as a higher histological score, characterized by subacute to chronic inflammatory cell infiltration, erosion or occasional ulcer formation, and crypt loss (DSS 12.33±0.33 vs. Normal 0.67±0.21; *P*<0.0001) (Figs. 2A–C). Treatment with 5-ASA (7.17±0.65), as well as with zonarol at 10 mg/kg (7.33±0.95) and 20 mg/kg (5.40±1.36) significantly reduced the inflammatory histological score on day 15 (*P*<0.0001, *P*<0.05, and *P*<0.05, respectively) (Fig. 2A–C). Moreover, the sum of the histological ulcer length in the mice in the zonarol 20 mg/kg group (0.73±0.14 cm) was significantly longer than that in the normal control mice (0.00±0.00 cm) on day 15 post-DSS administration (*P*<0.05), but was significantly shorter than that in the DSS positive control mice (3.68±1.25 cm) (*P*<0.05) (Fig. 2C). Indeed, the mice in the zonarol 20 mg/kg group had fewer and smaller colonic ulcers compared with the DSS positive control mice after DSS stimulation for 14 days (Fig. 2B). In contrast, the goblet cell number and size were not significantly between the untreated and each colitic group of mice, as confirmed by Alcian blue or PAS staining (data not shown).

### Zonarol represses inflammatory responses in the both subacute and chronic phases after DSS administration

The Gr-1-positive cells (accumulated neutrophils) in the mice in the zonarol 20 mg/kg group were significantly more decreased than in the positive control mice colons on day 15 post-DSS administration (DSS 259.7±48.7 per 10 fields vs. Zonarol 36.6±12.8 per 10 fields; *P*<0.05) (Fig. 3A). No, or fewer than five, neutrophils per 10 high-power fields were noted in sham-treated normal control mice (Normal 2.5±0.4 per 10 fields) (Fig. 3A).

IHC for CD3 demonstrated that the modestly-injured colons in mice in the zonarol 20 mg/kg group contained significantly fewer infiltrating T-lymphocytes, especially around the crypts in the lamina propria, compared with the DSS positive control mice on day 15 (DSS 473.8±39.3 per 10 fields vs. Zonarol 116.6±18.6 per 10 fields; *P*<0.0001) (Fig. 3B). Mac-2-staining also revealed significantly fewer macrophages per areas of mucosa in the mice in the zonarol 20 mg/kg group than in the mice in the positive control group in the present UC model (DSS 549.5±53.8 per 10 fields vs. Zonarol 65.4±9.1 per 10 fields; *P*<0.001) (Fig. 3C). Untreated normal control animals had markedly fewer chronic inflammatory cells (Figs. 3B–C), such as CD3^+^ T lymphocytes (64.0±3.0 per 10 fields) and Mac-2^+^ macrophages (28.8±4.2 per 10 fields) compared to the mice treated with zonarol at 20 mg/kg (*P*<0.05, respectively).

### Zonarol suppresses the expression of pro-inflammatory signaling molecules during DSS-induced UC

By performing an IF study, the TNF-α^+^ (*red-stained*) mucosal cells of the injured colons of the DSS-positive control mice were found to be much more common than those in the colons of the zonarol 20 mg/kg group (Fig. 4A). In addition, the numbers of both TNF-α^+^ (*red-stained*) and Mac-2^+^ (*green-stained*) macrophages in the lamina propria of the positive control mice were higher than those of the zonarol-treated mice 15 days after the DSS administration (Fig. 4A).

Next, we determined the levels of TNF-α and IL-6 in the serum by ELISA. Control mice had very low levels of expression, whereas TNF-α and IL-6 were significantly induced in the DSS-positive control mice. Zonarol significantly reduced the induction of TNF-α and IL-6 (TNF-α: Normal 3.85±0.67 pg/mL vs. DSS 8.23±1.21 pg/mL vs. Zonarol 3.66±1.24 pg/mL, *P*<0.05, respectively) (IL-6: Normal 5.85±2.08 pg/mL vs. DSS 36.54±8.98 pg/mL vs. Zonarol 5.57±2.23 pg/mL, *P*<0.05, respectively) (Fig. 4B).

Furthermore, IHC for iNOS revealed that, in mice with established DSS-induced UC, the iNOS expression level was overtly and substantially upregulated, especially in the surface colonic epithelium (Fig. 5A), but this was suppressed by zonarol treatments (Normal 0±0% vs. DSS 10.59±3.04% vs. Zonarol 2.28±1.20%, *P*<0.05, respectively) (Figs. 5A–B). No apparent iNOS-positive areas were seen in the untreated normal control mice.

### Zonarol decreases the apoptotic activity of the colonic epithelium in the DSS-induced UC model

Although a small, but substantial, number of apoptotic epithelial cells in crypts was observed in each group of mice 15 days after the DSS administration, the number of TUNEL^+^ large intestinal epithelial cells in the zonarol-treated mice was significantly higher than that in the normal control mice on day 15 post-DSS administration (*P*<0.05), but was significantly smaller than that in the DSS positive control mice (*P*<0.05) (Fig. 6A) (Normal 4.67±0.88 per 100 crypts vs. DSS 27.00±4.16 per 100 crypts vs. Zonarol 7.60±1.21 per 100 crypts; *P*<0.05, respectively). Double-IF staining (Fig. 6B) confirmed that these apoptotic cells (*green-stained* in the nuclei) were CK20^+^ colonic epithelial cells in the crypts (*red-stained* in the cytoplasm).

### Zonarol inhibits NO production without any effects on the survival of cultured RAW264.7 cells after LPS-stimulation

In order to confirm the absence of cytotoxicity at the concentrations used in the present *in vitro* study, we exposed RAW264.7 cells to different concentrations of zonarol and LPS (applied alone or in combination). After a 24 h incubation with different concentrations (0, 1, 2 and 5 µM) or zonarol, the viability of the cells was determined by performing a standard MTT assay. As shown in Figure 7A, the application of zonarol for 24 h at the indicated concentrations did not affect the viability of the cells, as indicated by the stable metabolic activity. Furthermore, the application of LPS (10 µg/mL), alone or together with different concentrations of zonarol, did not significantly affect the cell viability (Fig. 7A). Thus, none of the conditions used in the present study affected cell survival.

A hallmark of macrophage activation is the production of NO in response to LPS. Therefore, we determined whether or not zonarol could modulate the NO production in LPS-activated RAW264.7 cells by using the sister cultures used in the MTT assay. As shown in Figure 7B, LPS induced a strong increase in NO production in the RAW264.7 cells 24 h after LPS stimulation compared with the control level. Zonarol significantly suppressed the LPS-induced increase in NO in a concentration-dependent manner (Fig. 7B).

### Zonarol downregulates the expression of immune system mediators in cultured RAW264.7 cells after LPS stimulation

In order to confirm the inhibition of the hyperactivation of macrophages by zonarol, we examined the effects of the compound on other pro-inflammatory factors (IL-1β, IL-6 and iNOS) by performing RT-PCR (Fig. 8A–C, respectively). In the absence of LPS, the cells had very low expression levels of these genes; but in its presence, there was a significant increase in their expression levels. Zonarol at 2 µM modestly decreased the mRNA levels of IL-1β, IL-6 and iNOS (Fig. 8A–C, respectively).

## Discussion

The present study revealed, for the first time, a protective role for zonarol, a marine natural product isolated from *Dictyopteris undulata*, in a mouse model of UC. Seaweed zonarol significantly reduced the DSS-induced inflammation and apoptosis in Slc:ICR mice, very similar to 5-ASA treatments. Zonarol administration led to anti-inflammatory effects, including reduced bloody diarrhea, a decreased spleen/BW and less shortening of the colon length, a suppression of the extensive inflammatory and pro-inflammatory reactions and decreased apoptotic activity of the colonic epithelium in the large intestine. These pharmacological effects improved the DAI score, especially in the second half of day 9 to 15-post-DSS injury. Since UC is a chronic and idiopathic IBD mediated by various types of immune dysfuction [1], [2], zonarol might offer a promising therapeutic strategy and/or an alternative/additive therapy without any apparent adverse effects. We can also propose that DSS-induced apoptosis closely correlates with the potency of the inflammatory responses. Furthermore, we confirmed that the marine hydroquinone, zonarol, had anti-inflammatory actions in the RAW264.7 mouse macrophage cell line under *in vitro* LPS stimulation. However, our study has two limitations in its interpretation: the absence of a prevention administration of zonarol, as should be the case with nutraceuticals; and the model of colitis, which is a chemical but not immune model, even though it is an accepted one.

Polyphenols have received a great deal of attention, and are a category of compounds commonly used to treat various diseases, because they are present in teas, fruits and vegetables, and these play a pivotal role in anti-inflammatory responses due to their potent antioxidant effects [10], [12], [30]–[32]. Other groups have reported the protective roles of green tea and apple polyphenols against the same model of UC in mice [31], [32]. The administration of polyphenols significantly suppressed the DAI score, along with reducing the histological severity of colitis and downregulating the expression of pro-inflammatory signaling factors, such as TNF-α, IL-1β and IL-6 [31], [32]. Since our data regarding zonarol are novel in terms of the inhibition of UC by a marine bioproduct, zonarol may be useful as an alternative/additive herbal therapy (i.e., a complementary and alternative medicine (CAM)) for human UC. It is also conceivable that treatment with a marine natural product, zonarol, would have no severe side effects. Further follow-up investigations of the safety and efficacy of zonarol will be necessary before it can be considered for use against human IBDs.

Macrophages may play a key role in the DSS-induced UC via their expression of TNF-α. The current *in vivo* model showed chronic, but not acute, inflammatory cells-rich damaged mucosa consisting of a larger number of macrophages, rather than neutrophils and T-lymphocytes. Furthermore, TNF-α is the major and central key cytokine involved in initiating and perpetuating the colonic inflammatory responses in UC, and in fact, it has been recently reported that the most successful treatments for human IBDs are therapeutics targeting TNF-α [33], [34]. We herein used both an *in vivo* UC model and an *in vitro* macrophage cell line to assess the anti-inflammatory effects. Zonarol significantly suppressed the inflammatory reactions of the mouse macrophage cell line, RAW264.7, after stimulation by LPS, by downregulating the expression of various immune system mediators, including ILs and/or iNOS, and subsequently reducing the NO production. Consistent with these findings, the *in vivo* administration of the zonarol extract significantly decreased the levels of iNOS expression in the injured colonic surface epithelium, in addition to repressing various inflammatory and pro-inflammatory cytokines, such as TNF-α or IL-6. The NO system is an important signaling pathway associated with colonic inflammation, and is involved in strengthening the mucus barrier and potentially reducing the risk of subsequent tumorigenesis [35], [36]. Therefore, zonarol might reduce the risk of UC-associated cancer, even though zonarol treatments did not significantly affect the number or size of mucus-producing goblet cells in the present study. Further molecular and morphological studies are needed to clarify this possibility.

We herein demonstrated that zonarol inhibited the apoptosis of the colonic epithelium, possibly by interfering with a TNF-α-mediated signaling pathway. A smaller number of TUNEL^+^ colonic epithelial cells in the zonarol-treated mice was found compared to that in the DSS positive control mice. These results are also in line with findings of other groups, such as the finding that p53-upregulated modulator of apoptosis (PUMA) induction by TNF-α contributed to activating inflammation and epithelial apoptosis in the large intestine of a DSS-induced animal model of UC, and conversely, its suppression by anti-TNF-α antibody treatments prevented colitis and repressed the apoptotic activity of the colonic epithelium [37]. In fact, anti-TNF-α therapies for treating human IBD patients have been revealed to inhibit not only the inflammatory responses, but also epithelial cell apoptosis [37], [38]. Apoptosis has been considered to be one of major hallmarks in strongly regulating homeostatic and pathogenic mechanisms of intestinal epithelium in IBDs, even though the molecular basis of epithelial apoptosis in response to aberrant intestinal inflammation is unclear [39]. On the other hand, apoptosis in the colonic epithelium is responsible for disrupting the mucosal integrity and barrier function to bacterial invasion [37]–[39]. In these senses, it remains to be elucidated whether apoptosis or inflammation comes first in the development of UC, reminiscent of the chicken-and-egg problem. Taken together, our present data showed, for the first time, that a marine hydroquinone can play a pivotal role in protecting against both colitis and apoptosis, decreasing the activity of UC, at least partially by inhibiting the TNF-α signaling pathway.

In conclusion, based on our collected data, we believe that zonarol plays critical and broad functional roles in ameliorating DSS-induced colonic injury (i.e., improves the DAI score), together with (i) reducing the accumulation and recruitment of various inflammatory cells into the modestly damaged mucosa, accompanied by downregulation of the expression of various inflammatory and pro-inflammatory mediators; (ii) in particular, by decreasing the number of mucosal TNF-α-expressing macrophages; (iii) suppressing the apoptosis of the intestinal epithelium. This was strongly supported by (iv) the *in vitro* experiments, showing that zonarol alleviated the inflammatory responses of the mouse RAW264.7 cells after stimulation by LPS. Hence, we can conclude that zonarol prevents the development of subacute (induced by macrophages, T-lymphocytes, and to a lesser extent neutrophils) to chronic (predominantly induced by macrophages and T-lymphocytes) inflammatory responses and the subsequent apoptotic activity, exerting various beneficial effects in the DSS-induced mouse model of UC. All of these features indicate that the oral administration of the marine hydroquinone, zonarol, from *Dictyopteris undulata* might offer an alternative/additive therapeutic strategy (i.e., CAM) against human IBDs, including UC.

## Supporting Information

Figure S1 **A schematic presentation of the experimental procedure in DSS-induced mice UC model.** DSS: dextran sulfate sodium, 5-ASA: 5-aminosalicylic acid, DAI: disease activity index, H&E: hematoxylin and eosin, IHC: immunohistochemistry, IF: immunofluorescence.
(PPTX)

Figure S2 **The separation scheme for compound 1.** The MeOH extract from the seaweed *Dictyopteris undulate* showed anti-edematous activity in mice. Bioassay-guided fractionation of the crude extract (108.0 g as 100%) gave compound 1 (1.1% of yield), which had the activity. MeOH: methanol, HPLC: high performance liquid chromatography, ODS: Octadecyl Silyl.
(PPTX)

Figure S3 **Inhibitory effects of purified zonarol in a carrageenan-induced paw edema mouse model.** **A**) An increased paw edema volume. **B**) The increase in paw edema thickness. Open circles: control. Closed circles: zonarol (62.5 mg/kg) administration. Each value represents the mean ± SE. (n = 5 mice per group). *P<0.05 vs control.
(PPTX)

Figure S4 **The NMR spectral data of purified zonarol.** **A**) The proton NMR data (400 MHz, MeOD). **B**) The carbon NMR data (100 MHz, MeOD). NMR: nuclear magnetic resonance.
(PPTX)
