# Supplementary material for: Marine Hydroquinone Zonarol Prevents Inflammation and Apoptosis in Dextran Sulfate Sodium-Induced Mice Ulcerative Colitis
Source: PLoS One. 2014 Nov 19;9(11):e113509. doi: 10.1371/journal.pone.0113509 (PMC4237432; doi:10.1371/journal.pone.0113509)
Supplement: Figure S1 — A schematic presentation of the experimental procedure in DSS-induced mice UC model. DSS: dextran sulfate sodium, 5-ASA: 5-aminosalicylic acid, DAI: disease activity index, H&E: hematoxylin and eosin, IHC: immunohistochemistry, IF: immunofluorescence. (PPTX) [file pone.0113509.s001.pptx]

## Slide 1
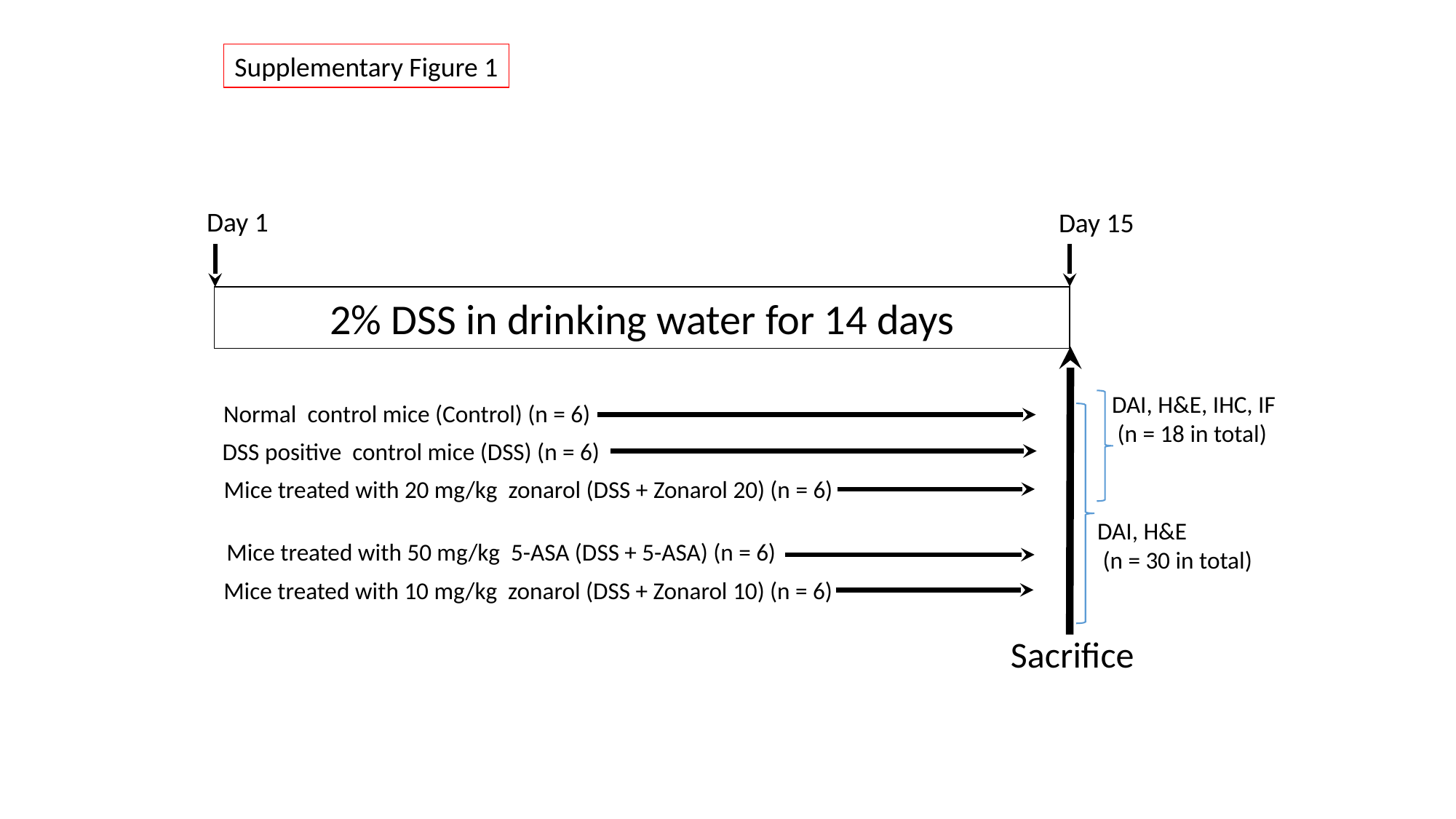

Supplementary Figure 1
Day 1
Day 15
2% DSS in drinking water for 14 days
DAI, H&E, IHC, IF
 (n = 18 in total)
Normal control mice (Control) (n = 6)
DSS positive control mice (DSS) (n = 6)
Mice treated with 20 mg/kg zonarol (DSS + Zonarol 20) (n = 6)
DAI, H&E
 (n = 30 in total)
Mice treated with 50 mg/kg 5-ASA (DSS + 5-ASA) (n = 6)
Mice treated with 10 mg/kg zonarol (DSS + Zonarol 10) (n = 6)
Sacrifice
