# Supplementary material for: Marine Hydroquinone Zonarol Prevents Inflammation and Apoptosis in Dextran Sulfate Sodium-Induced Mice Ulcerative Colitis
Source: PLoS One. 2014 Nov 19;9(11):e113509. doi: 10.1371/journal.pone.0113509 (PMC4237432; doi:10.1371/journal.pone.0113509)
Supplement: Figure S2 — The separation scheme for compound 1. The MeOH extract from the seaweed Dictyopteris undulate showed anti-edematous activity in mice. Bioassay-guided fractionation of the crude extract (108.0 g as 100%) gave compound 1 (1.1% of yield), which had the activity. MeOH: methanol, HPLC: high performance liquid chromatography, ODS: Octadecyl Silyl. (PPTX) [file pone.0113509.s002.pptx]

## Slide 1
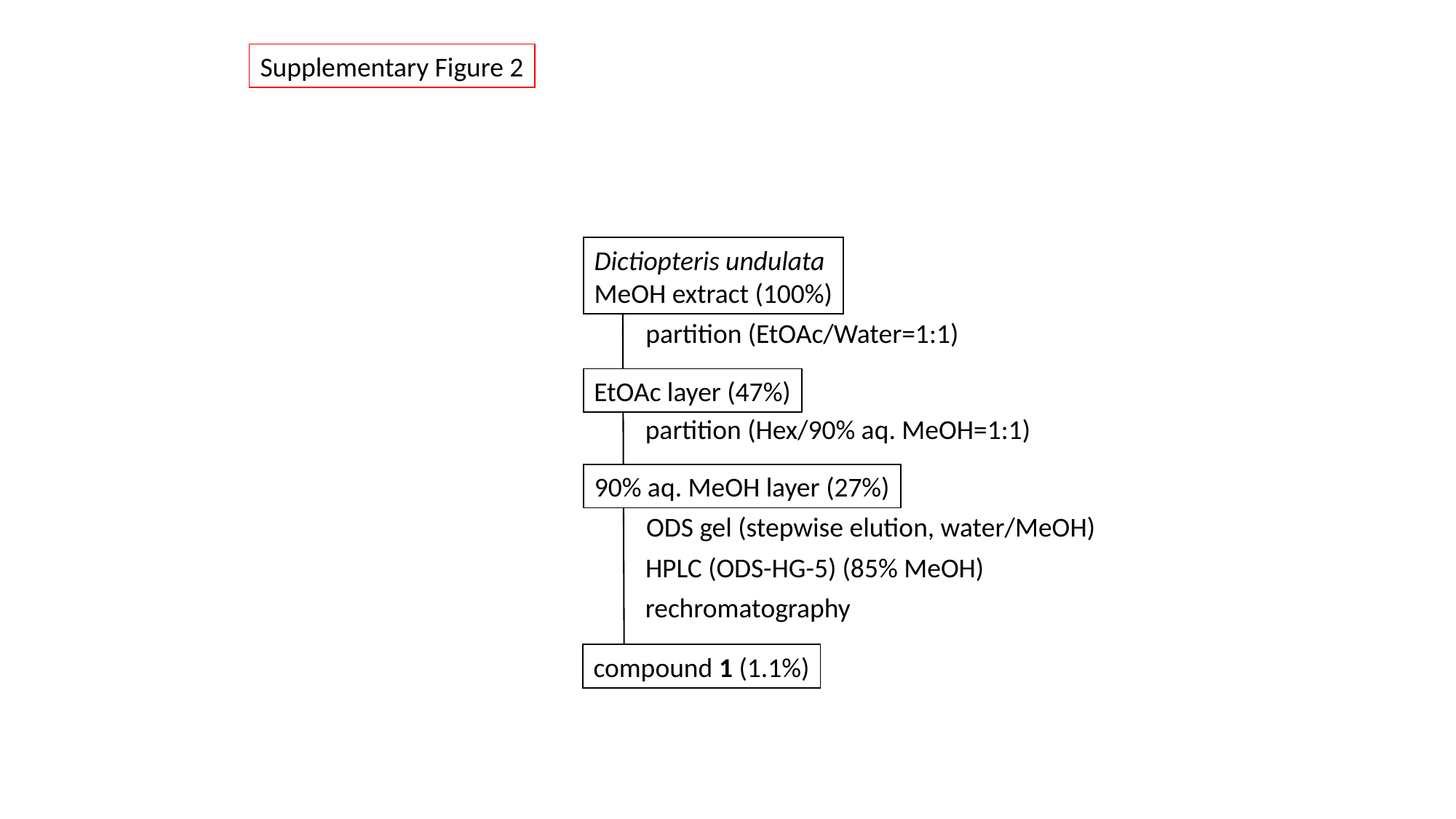

Supplementary Figure 2
Dictiopteris undulata
MeOH extract (100%)
partition (EtOAc/Water=1:1)
EtOAc layer (47%)
partition (Hex/90% aq. MeOH=1:1)
90% aq. MeOH layer (27%)
ODS gel (stepwise elution, water/MeOH)
HPLC (ODS-HG-5) (85% MeOH)
rechromatography
compound 1 (1.1%)
