# Supplementary figures and images for: Marine Hydroquinone Zonarol Prevents Inflammation and Apoptosis in Dextran Sulfate Sodium-Induced Mice Ulcerative Colitis
Source: PLoS One. 2014 Nov 19;9(11):e113509. doi: 10.1371/journal.pone.0113509 (PMC4237432; doi:10.1371/journal.pone.0113509)

## Slide 1
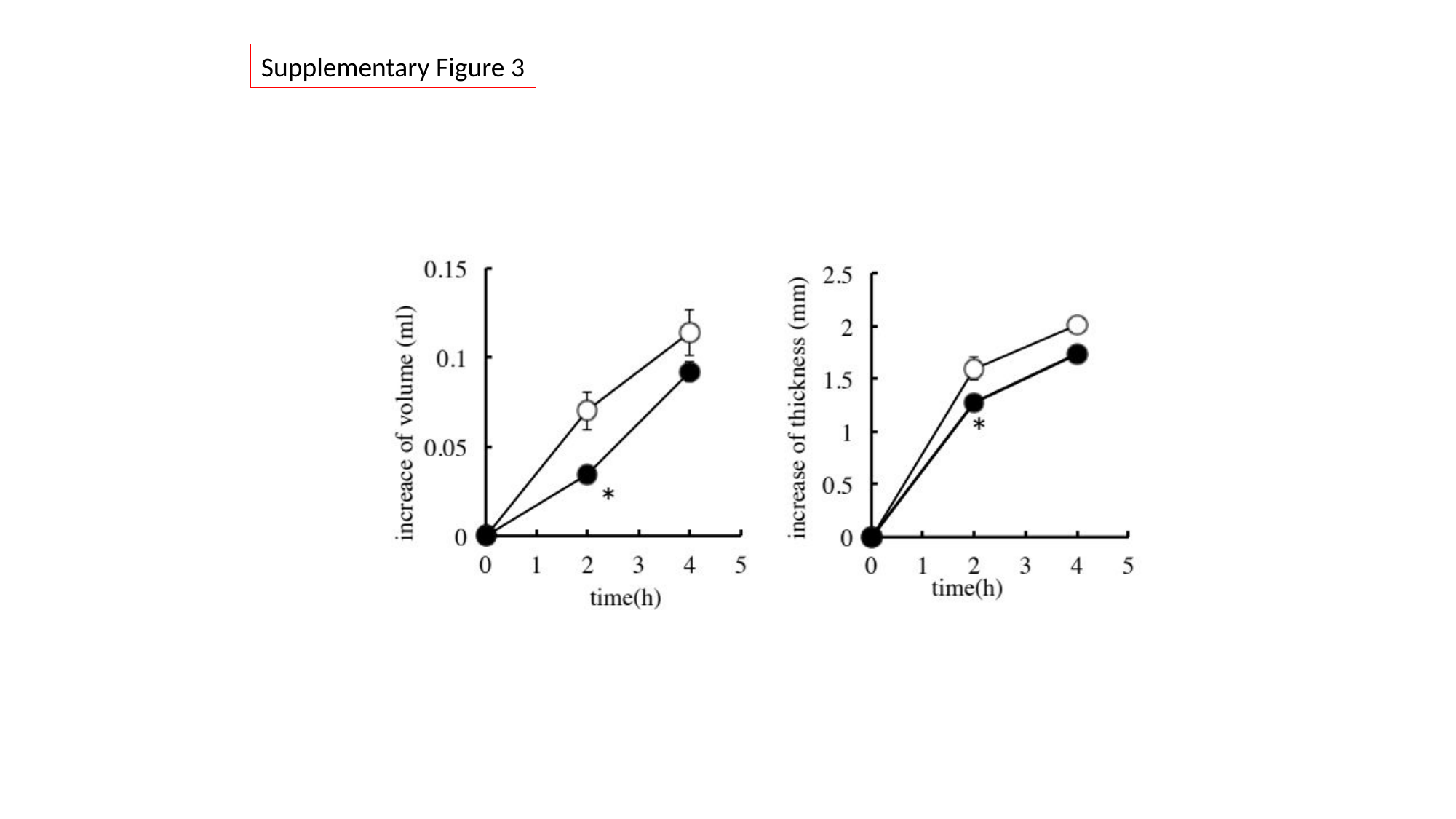

Supplementary Figure 3

Supplement: Figure S3 — Inhibitory effects of purified zonarol in a carrageenan-induced paw edema mouse model. A) An increased paw edema volume. B) The increase in paw edema thickness. Open circles: control. Closed circles: zonarol (62.5 mg/kg) administration. Each value represents the mean ± SE. (n = 5 mice per group). *P<0.05 vs control. (PPTX) [file pone.0113509.s003.pptx]

## Slide 1
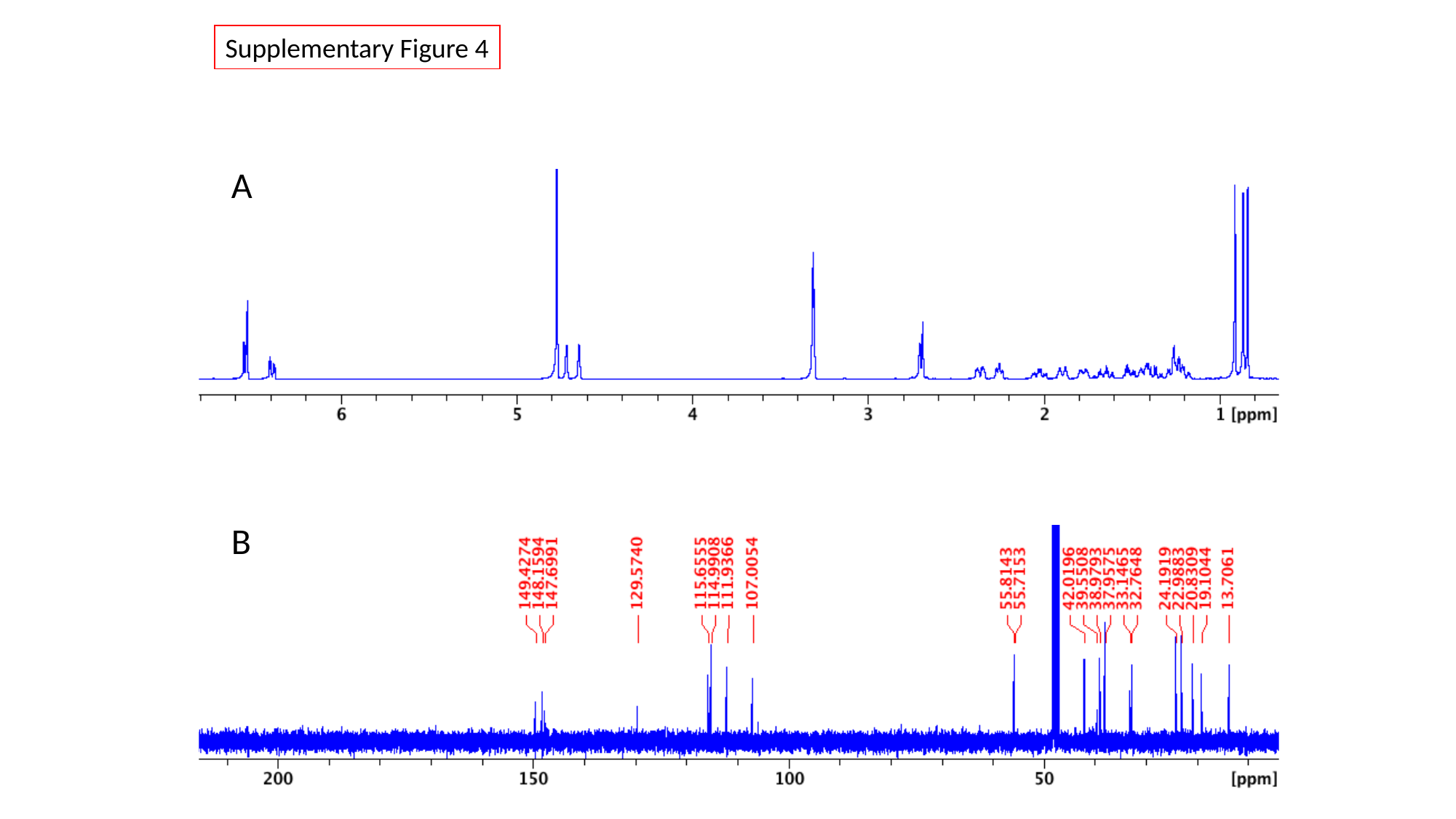

Supplementary Figure 4
A
B

Supplement: Figure S4 — The NMR spectral data of purified zonarol. A) The proton NMR data (400 MHz, MeOD). B) The carbon NMR data (100 MHz, MeOD). NMR: nuclear magnetic resonance. (PPTX) [file pone.0113509.s004.pptx]
